# Protein Kinase R Modulates c-Fos and c-Jun Signaling to Promote Proliferation of Hepatocellular Carcinoma with Hepatitis C Virus Infection

**DOI:** 10.1371/journal.pone.0067750

**Published:** 2013-07-02

**Authors:** Takao Watanabe, Yoichi Hiasa, Yoshio Tokumoto, Masashi Hirooka, Masanori Abe, Yoshio Ikeda, Bunzo Matsuura, Raymond T. Chung, Morikazu Onji

**Affiliations:** 1 Department of Gastroenterology and Metabology, Ehime University Graduate School of Medicine, Toon, Ehime, Japan; 2 Gastrointestinal Unit, Massachusetts General Hospital and Harvard Medical School, Boston, Massachusetts, United States of America; University of Modena & Reggio Emilia, Italy

## Abstract

Double-stranded RNA-activated protein kinase R (PKR) is known to be upregulated by hepatitis C virus (HCV) and overexpressed in hepatocellular carcinoma (HCC). However, the precise roles of PKR in HCC with HCV infection remain unclear. Two HCV replicating cell lines (JFH-1 and H77s), generated by transfection of Huh7.5.1 cells, were used for experiments reported here. PKR expression was modulated with siRNA and a PKR expression plasmid, and cancer-related genes were assessed by real-time PCR and Western blotting; cell lines were further analyzed using a proliferation assay. Modulation of genes by PKR was also assessed in 34 human HCC specimens. Parallel changes in c-Fos and c-Jun gene expression with PKR were observed. Levels of phosphorylated c-Fos and c-Jun were upregulated by an increase of PKR, and were related to levels of phosphorylated JNK1 and Erk1/2. DNA binding activities of c-Fos and c-Jun also correlated with PKR expression, and cell proliferation was dependent on PKR-modulated c-Fos and c-Jun expression. Coordinate expression of c-Jun and PKR was confirmed in human HCC specimens with HCV infection. PKR upregulated c-Fos and c-Jun activities through activation of Erk1/2 and JNK1, respectively. These modulations resulted in HCC cell proliferation with HCV infection. These findings suggest that PKR-related proliferation pathways could be an attractive therapeutic target.

## Introduction

Hepatitis C virus (HCV) is a leading cause of chronic liver disease and is a leading indication for liver transplantation [1]. HCV establishes persistent infection and induces chronic hepatitis, which leads to liver cirrhosis (LC) and, frequently, to hepatocellular carcinoma (HCC) [2]. However, the precise mechanisms involved in the induction of heptocarcinogenesis by HCV, and details of viral effects on tumor progression, remain unclear.

Of the many cellular proteins stimulated by HVC replication, double-stranded RNA-activated protein kinase R (PKR) appears to play a key antiviral role [3]. Double stranded-RNA produced by RNA viral replication is known to be a potent activator of PKR [4]. Activated PKR, in turn, induces PKR phosphorylation, and then PKR dimerizes and phosphorylates eukaryotic initiation factor-2 alpha (eIF2α), which inhibits protein synthesis, including that of virally-encoded proteins [5]. Moreover, PKR is recognized as a key arm of the antivirus and antiproliferative effects of interferon, the most important clinically active agent against HCV [3]. PKR plays multiple roles in cell growth, differentiation, apoptosis, and responses to cellular stress occurring during RNA virus infections [6].

The HCV generates dsRNA during the process of viral replication, which is thought to activate PKR and then induce the host antiviral responses [7]. Several reports have indicated that PKR can directly inhibit HCV replication [7]–[9]. We previously reported that PKR was overexpressed and activated in HCC with HCV infection, as compared with surrounding non-HCC tissues [10]. Moreover, the level of HCV-RNA was detected and reduced in HCC compared with surrounding non-HCC tissue, indicating that the overexpressed PKR in HCC tissues retains its antiviral function against HCV [10].

PKR was originally thought to function as a tumor suppressor protein, as it induces the apoptotic response [11], [12], and it was suggested that PKR inhibits cell growth and proliferation [13], [14]. However, results of further studies demonstrating functioning PKR in HCC with HCV infection [10] suggest that it could act as a tumor stimulator rather than as a suppressor.

The aim of this study was to identify the roles of PKR in HCC with HCV infection, and to evaluate whether overexpressed PKR in HCC has beneficial or malignant effects in patients with this disease.

## Materials and Methods

### Cell Culture and Transfection

The hepatoma cell line Huh 7.5.1 (kindly provided by Dr. Francis V. Chisari, Department of Immunology and Microbial Science, The Scripps Research Institute, La Jolla, CA, USA) was grown and maintained in Dulbecco’s modified Eagle’s medium (DMEM) (Life Technologies, Carlsbad, CA, USA) supplemented with 10% fetal bovine serum (FBS) (Life Technologies) and 1% penicillin. Cells were maintained at 37°C in a humidified atmosphere of 5% CO_2_ and 95% air, and the culture medium was changed three times per week.

HCV-infected HCC cell lines used were JFH1 and H77s. JFH1 cells were generated by transfection of Huh 7.5.1cells with HCV-RNA synthesized by transcription from pJFH1-full (kindly provided by Dr. Takaji Wakita, National Institute of Infectious Diseases, Tokyo, Japan), which encodes the HCV genotype 2a sequence [15]. For the HCV-RNA transfection, Huh 7.5.1 cells were resuspended in Opti-MEM I (Life Technologies) containing 10 µg of synthesized HCV-RNA, and were subjected to an electric pulse (960 µF, 260 V) using the Gene Pulser II apparatus (Bio-Rad, Richmond, CA, USA). H77s cells were made by transfection with HCV-RNA transcripted from pH77s-full (kindly provided by Dr. Stanley M. Lemon, University of North Carolina at Chapel Hill, Chapel Hill, NC, USA), which encodes the HCV genotype 1a sequence [16], [17]; the transfection procedure was the same as that previously described.

### Patients and Liver Specimens

HCC specimens were obtained from patients who underwent surgery at our institution from 2007 to 2012. Written informed consent was obtained from all patients. The study protocol conformed to the ethical guidelines of the Declaration of Helsinki, and was approved by the Institutional Review Board at Ehime University Hospital (Approval No. 0710004). This study was registered by the University Hospital Medical Information Network (UMIN) Clinical Trials Registry (registration number 000008652). Table 1 lists clinicopathological features of the 34 HCC patients enrolled. Among the 34 specimens, 17 were from patients with HCV.

**Table 1:** Clinicopathological parameters of patients with hepatocellular carcinoma (HCC) (n = 34).

	Low PKR (n = 17)	High PKR (n = 17)	p-value
**Age, years (range)**	65 (36–82)	70 (49–79)	0.245
**Gender (M/F)**	14/3	13/4	1.000
**Virus (HBV/HCV/None)**	7/7/3	4/10/3	0.510
**Child-Pugh (A/B/C)**	15/2/0	17/0/0	0.145
**Fibrosis (F1/F2/F3/F4)**	2/3/3/9	2/3/3/9	1.000
**Pathological stage (1/2/3/4)**	6/6/3/1	2/9/6/0	0.206
**AFP, ng/mL (range)**	6.0 (1.6–1525)	25 (0–7720)	0.435
**DCP, mAU/mL (range)**	65 (13–418820)	212(16–45133)	0.356
**PT, % (range)**	86.9 (56–181)	85.10 (73–129)	0.790
**Tumor size**	24 (10–160)	35 (20–75)	0.816
**Tumor differentiation (well/moderate/poor)**	3/11/3	4/13/0	0.191
**Tumor multiplicity (solitary/multiple)**	13/4	13/4	1.000

HBV, hepatitis B virus; HCV, hepatitis C virus; AFP, alpha-fetoprotein; DCP, des-carboxyprothrombin; PT, prothrombin time.

Samples of freshly resected liver specimens were incubated with RNAlater (Life Technologies) overnight at 4°C, and were then frozen and stored at −80°C until used. Additional samples were placed on dry ice and frozen immediately, and then stored at −80°C until used for protein extraction.

### RNA Interference and PKR Inhibitor Assay

A PKR-specific siRNA (GAG AAU UUC CAG AAG GUG A, nt. 584–604) was designed using a PKR sequence template (accession number NM002759), and was then synthesized by Thermo Fisher Scientific (Waltham, MA, USA) [18]. Control siRNA was obtained from Cosmo Bio (Tokyo, Japan); c-Fos siRNA and c-Jun si-RNA were obtained from Thermo Fisher Scientific. Huh 7.5.1, JFH1 and H77s cell lines at 50% confluence in 6-well plates were transfected with 50 pM siRNA using RNAiMax (Life Technologies). For the assay with a PKR inhibitor, JFH1 or H77s cells were incubated with 300 nM of PKR inhibitor (Merck, Darmstadt, Deutschland) for 24 h.

### PKR Plasmids

Plasmids encoding PKR genes were kindly provided by Dr. Michael Gale, Jr. (University of Washington, Seattle, WA, USA) [4]. The plasmid pOS8, which expresses β-galactosidase, was used as the control plasmid [19]. Each plasmid (1 µg/mL) was transfected into Huh 7.5.1, JFH1, and H77s at 50% confluence in 6-well plates using Lipofectamine 2000 (Life Technologies).

### RNA Extraction, cDNA Synthesis and Real-time RT-PCR

Total RNA was extracted with TRIzol reagent (Life Technologies). Reverse transcription was carried out using the RT-PCR kit (Applied Biosystems, Foster City, CA, USA), and cDNA was quantified by real-time PCR using LightCycler technology and SYBR green I dye (Roche Diagnostics, Mannheim, Germany). Real-time PCR for PKR was performed with 2 µl of purified cDNA in a reaction SYBR green mixture containing 4 mM MgCl_2_, and 5 pM each of forward primer (5′-AGCACACTCGCTTCTGAATC-3′) and reverse primer (5′-CTGGTCTCAGGATCATAATC-3′). PCR consisted of an initial denaturation step for 10 min at 95°C, then 40 cycles under the following conditions: 10 sec at 95°C, 10 sec at 58°C, and 15 sec at 72°C. For PCR amplification of GAPDH, c-Fos, c-Jun, and IL-8, commercial primer sets (Roche Search LC, Heidelberg, Germany) were used under conditions recommended by the manufacturer. PCR amplification of HCV RNA proceeded as described elsewhere [20]. The relative mRNA expression levels of target host genes were defined by dividing by the amount of GAPDH mRNA and were then evaluated by statistical analysis.

### PCR Array Analysis

For comprehensive analysis of the role of PKR, the RT2 Profiler PCR array system (QIAGEN, Tokyo, Japan) and the LightCycler system (Roche) were used according to the manufacturers’ instructions. Threshold cycle values were analyzed using web-based PCR array data analysis software found at http://www.sabiosciences.com/pcr/arrayanalysis.php. Of the kits supplied with the PCR array system, the Cancer PathwayFinder PCR array was used. Before applying the data, it was determined that the reverse transcriptase control, cDNA control, and positive PCR control levels were within the accepted range.

### Western Blotting

Proteins were extracted with RIPA buffer comprising 10 mmol/L Tris, PH 7.4, 150 mmol/L NaCl, 0.5% v/v NP-40 and 1% w/v sodium dodecyl sulphate (SDS). For Western immunoblotting, 20 µg of protein were applied to lanes of 4–12% Bis-Tris Gels (Life Technologies), then blotted onto Immobilon-P membranes (Millipore, Bedford, MA, USA) and incubated with the relevant antibody: anti-beta-actin (Chemicon, Temecula, CA, USA), anti-PKR (product number: 3210), anti-eIF2α (5324), anti-phospho-eIF2α (3398), anti-Erk1/2 (4695), anti-phospho-Erk1/2 (4370), anti-JNK (9252), anti-phospho JNK (4668), anti-c-Jun (9165), anti-phosphor-c-Jun (3270), anti-c-Fos (2250) (Cell Signaling, Danvers, MA, USA), anti-phospho PKR (Life Technologies) or anti-phospho c-Fos (Abcam, Tokyo, Japan) antibody. Appropriate species-specific conjugated secondary antibody kits were commercially obtained (General Electric, Charles Coffin, NY, USA). Proteins were detected using the ECL Prime Kit or the ECL Kit (General Electric). Bands were quantified by normalization to β-actin using Image J Software (National Institutes of Health Bethesda, MD, USA).

### Cell Proliferation Assay

Cell viability was quantified by a MTS assay (Promega, Fitchburg, WI, USA). After 24 h of siRNA or plasmid transfection in 6-well plates, cells were resuspended and then 2 ×10^3^ cells were seeded in 96-well plates. At each time point, cells were treated with MTS reagent and incubated for 120 min. Absorbance at 450 nm was recorded. U0126 (Merck), a selective inhibitor of MAP kinase kinase (MEK), was used to inhibit the c-Fos signaling pathway, and SP600125 (Merck), a selective inhibitor of JNK, was used to inhibit the c-Jun signaling pathway.

### Enzyme-linked Immunosorbent Assay for IL-8 and HCV Core Protein

Concentrations of IL-8 were measured in culture supernatant fluids using an enzyme-linked immunosorbent assay (ELISA) kit (R&D Systems, Minneapolis, MN, USA). The lower limit of detection for IL-8 was 31.2 pg/mL. HCV core antigen in cell lysates was quantified using HCV core antigen ELISA kits (Ortho-Clinical Diagnostics, Raritan, NJ, USA) following the manufacturer’s instructions. The lower limit of detection for HCV was 44.4 fmol/L.

### Luciferase Assays

Cells were transfected with a wild type IL-8 promoter conjugated to firefly luciferase reporter constructs (pIL-8) provided by Dr. Charalabos Pothoulakis (University of California, Los Angeles, CA, USA) [21], together with a control reporter plasmid conjugated with Renilla luciferase reporter constructs, phRL-TK^Int−^ (Promega). Luciferase activities in the samples were measured 48 h later using the Dual-Luciferase Reporter Assay System (Promega) and a luminometer (Microtec, Funabashi, Chiba, Japan). The level of transcription was evaluated as the ratio of firefly luciferase to Renilla luciferase.

### Wound Healing Assay

JFH1 or H77s cells (2×10^5/^well) were seeded into 6-well plates and incubated for two days. After confirming that a complete monolayer had formed, the monolayers were wounded by scratching lines onto the cultures using a plastic tip. The wells were then washed once to remove any debris, and observed and photographed under the microscope. Thereafter, the plates were incubated at 37°C in 5% CO2 for 48 h, after which the cells were observed and photographed. The distance that the cells had migrated was measured on the photomicrographs [22]. The percent wounded area filled was calculated as follows: {(mean wounded breadth – mean remaining breadth)/mean wounded breadth}×100.

### c-Fos and c-Jun Transcription Assay

The c-Jun and c-Fos transcription assay was performed using TransAM™ (Active Motif, Carlsbad, CA, USA) according to the manufacturer’s instructions, to detect the DNA binding capacity of c-Jun or c-Fos containing AP-1 dimers. This assay is equivalent to the Electrophoresis Mobility Shift Assay (EMSA) used to evaluate DNA binding capacity [23]. Briefly, JFH1 cells were treated with PKR siRNA or control siRNA, and after 48 h, 10 µg of nuclear extract (containing activated transcription factor) was added to oligonucleotide-coated wells (containing 5′-TGAGTCA-3′). After 20 min of incubation at room temperature with mild agitation, the plate was washed, and then incubated with 100 µL/well of diluted anti-c-Jun or anti-c-Fos antibody for 1 h. The plate was washed three times and 100 µl HRP-conjugated antibody was added for 1 h. Developing solution was added for 10 min. The reaction was stopped and absorbance at 450 nm was determined.

### Statistical Analysis

All statistical analyses were performed using SPSS 14.0 software (SPSS, Chicago, IL, USA). Data are expressed as mean ± SEM. Statistical differences were determined using the Mann-Whitney U test. The relationships between c-Fos and c-Jun were expressed as Pearson product-moment correlation coefficients. P-values < 0.05 were considered to be significant.

## Results

### PKR Knockdown and Upregulation by PKR siRNA and PKR-encoding Plasmid in HCV-infected HCC Cells

**Figure 1:**
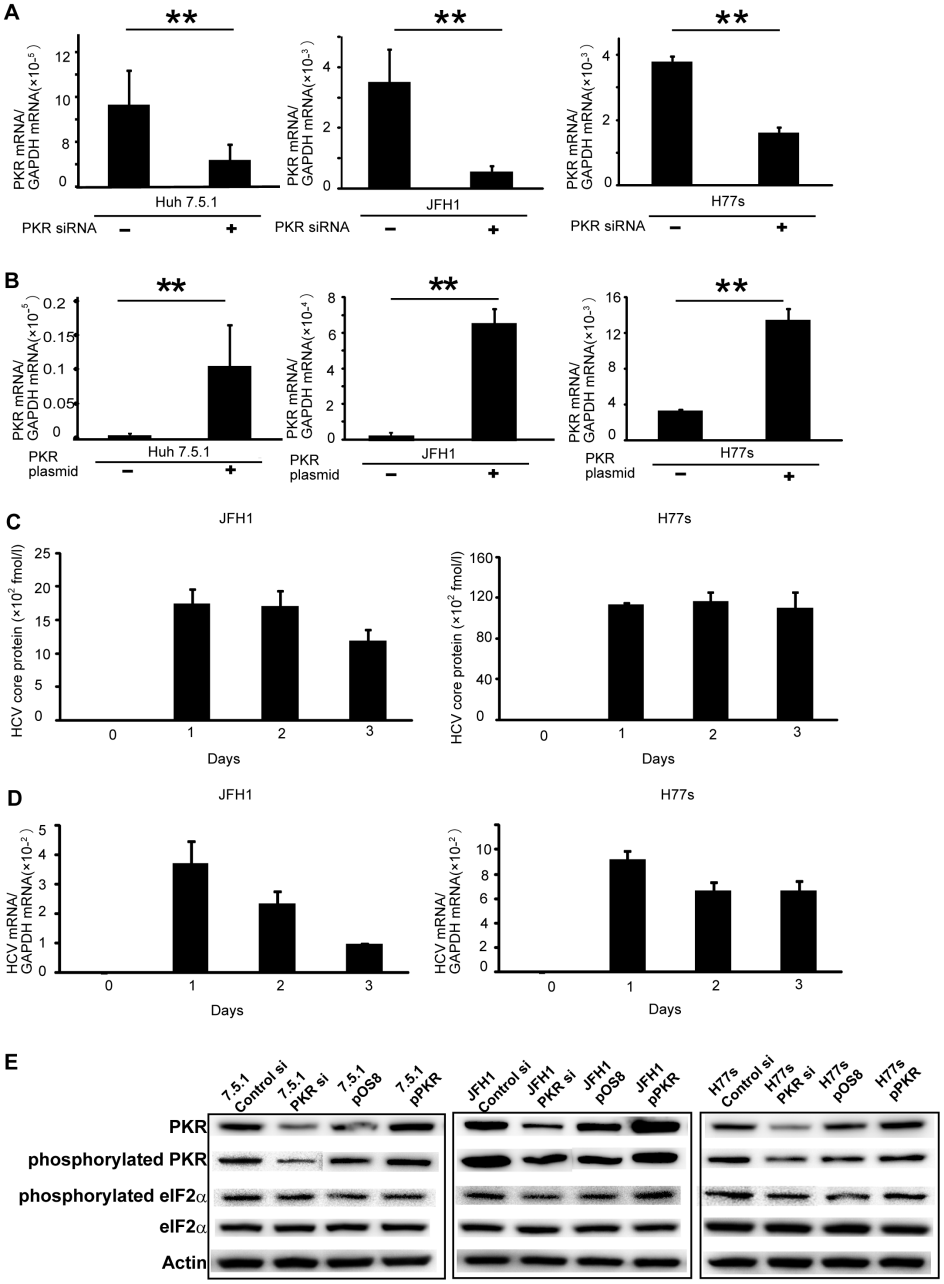
Down- and upregulation of PKR gene expression by PKR siRNA or by a PKR-expression plasmid, respectively, in liver cancer cell lines, Huh 7.5.1, JFH1 and H77s. Cells were transfected with either PKR siRNA or control siRNA, then PKR mRNA was quantified by real-time RT-PCR; PKR was knocked down (A). Cells were transfected with PKR-expression plasmid (pPKR) or control plasmid (pOS8), then subjected to real-time RT-PCR; PKR mRNA was upregulated by pPKR (B). Mean ± SEM of six replicates. **p<0.01. The amount of HCV core protein in JFH1 and H77s cells were measured by ELISA. HCV core protein was stably expressed in both cell types at least three days after transfection with the HCV-RNA (C). The amount of HCV mRNA in JFH1 and H77s cells was measured by real-time RT-PCR. HCV RNA was expressed in both cell types at least three days after transfection with the HCV-RNA (D). Mean ± SEM of four replicates. PKR and phosphorylated PKR protein expression evaluated by Western blotting; results confirmed PKR mRNA data (E).

PKR siRNA was designed to knock down PKR gene expression in liver cancer cell lines [18]. As shown by real-time RT-PCR, PKR siRNA efficiently knocked down PKR mRNA levels in Huh7.5.1, JFH1 and H77s cell lines by day 2 after transfection (Fig. 1A). Use of this method also revealed that the PKR-encoding plasmid (pPKR) efficiently upregulated PKR mRNA in experimental cell lines by day 2 after transfection (Fig. 1B).

The amount of HCV core protein was measured before (day 0) and after (days 1–3) transfection with HCV-RNA from pJFH1-full or pH77s-full. The HCV core protein expressed stably at least three days after the transfection in both JFH1 and H77s cells (Fig. 1C). The amount of HCV-RNA was also evaluated by real-time RT-PCR at the time core protein was measured in JFH1 and H77s cells (Fig. 1D). The level of HCV-RNA in JFH1 cells on day 3 was decreased, which may reflect the near-confluent cell culture condition, as reported previously [15]. However, HCV-RNA was expressed in both cell types at least three days after transfection.

Confirming results reported previously [7], HCV infection enhanced PKR activation, indicating upregulation of phosphorylated PKR, as shown in Fig. S1. Western blotting demonstrated the expected corresponding changes in PKR and phosphorylated PKR protein expression (Fig. 1E). To evaluate the influence of PKR activation, the expression of phosphorylated eIF2α was also determined. In the HCV-infected cells (JFH1 and H77s), the expression of phosphorylated eIF2α was altered by the knockdown or overexpression of PKR; however, in uninfected Huh 7.5.1 cells, the expression of phosphorylated eIF2α was not significantly altered (Fig. 1E). The expression of PKR, phosphorylated PKR, and phospohorylated eIF2α was quantified by normalizing to expression of β-actin using Image J Software (Fig. S2).

### PKR Upregulates the AP-1 Family Transcription Factors c-Fos and c-Jun in HCV-infected HCC Cells

**Figure 2:**
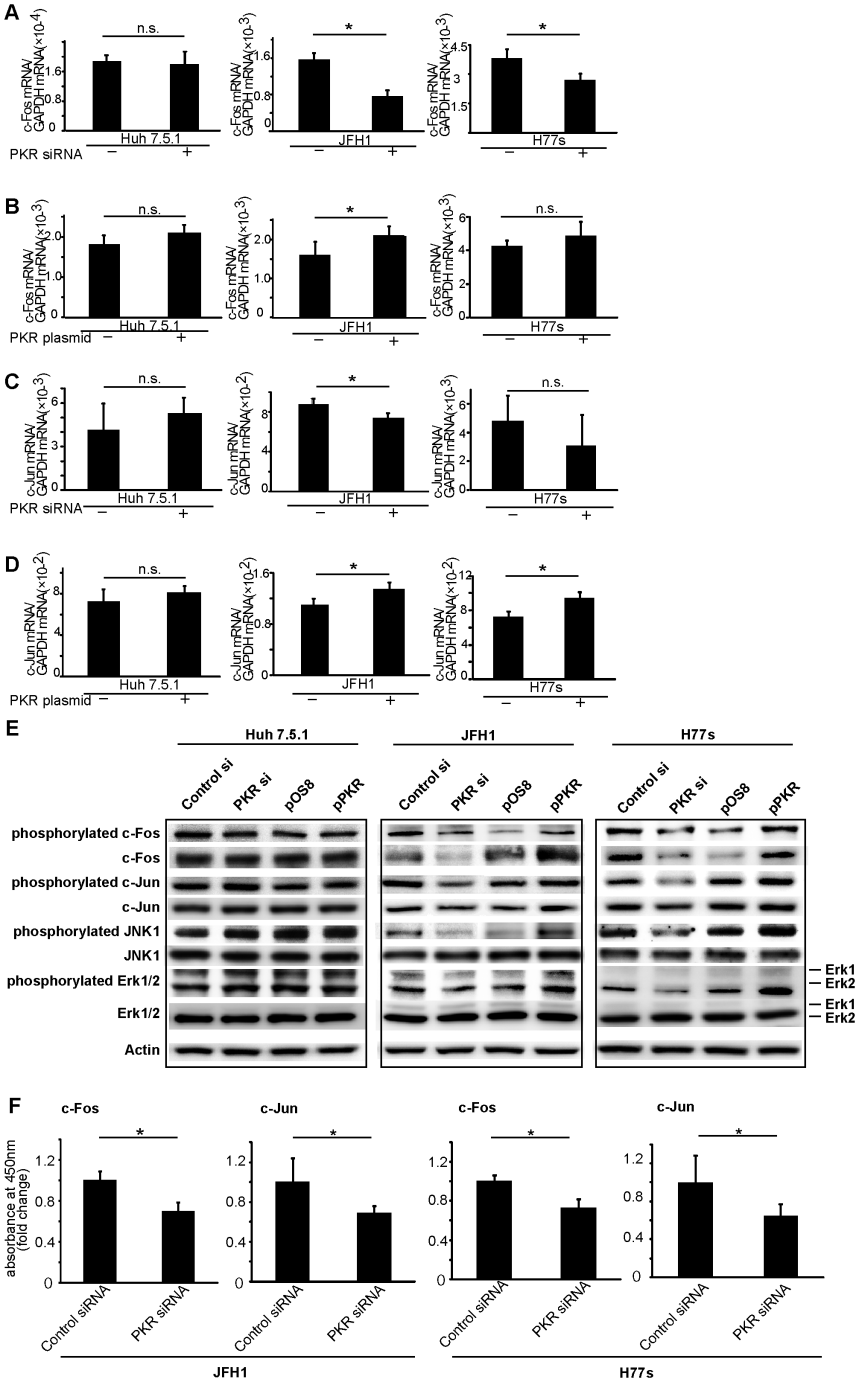
PKR upregulates c-Fos and c-Jun mRNA and protein in the HCC cell lines with HCV infection, JFH1 and H77s. Cells were transfected with either control or PKR siRNA, and with either control or PKR-expression plasmid (pPKR). c-Fos mRNA was decreased by PKR knockdown (A), and c-Fos was upregulated by PKR overexpression (B). c-Jun mRNA was downregulated by PKR siRNA (C), and upregulated by pPKR (D). Mean ± SEM of six replicates. *p<0.05. n.s.: not significant. Protein expression of c-Fos, c-Jun, JNK1, Erk and their phosphorylated forms determined by Western blotting. Bothc-Fos and c-Jun signaling pathways were activated by PKR (E). DNA binding activities of c-Jun and c-Fos were downregulated by PKR knockdown (F). Mean ± SEM of six replicates. *p<0.05.

Use of the Cancer Pathway Finder PCR array indicated that several cancer pathways were modulated by up- or downregulation of PKR (Table S1). AP-1 family transcription factors, including c-Fos and c-Jun, were modulated in JFH1 cells, and the modulation was more evident in JFH1 than in uninfected Huh7.5.1 cells. Additionally, the interleukin IL-8 gene, which is a downstream AP-1 entity, was also altered by the PKR in JFH1 cells. These results indicated that the modulation of c-Fos and c-Jun by PKR was more evident because the function of PKR was enhanced by HCV infection. Real-time RT-PCR analysis of JFH1 confirmed modulation of c-Fos and c-Jun genes due to the expression of PKR with knockdown by PKR siRNA and with overexpression by PKR plasmid (Fig. 2A–D). Similar trends were observed in H77s, although some results were not statistically significant (Fig. 2A–D).

Alteration of the MAPK pathways, including of c-Fos and c-Jun, by PKR in the HCV-infected HCC cells became the next focus of investigation. Using Western blot analysis, the effect of modulation of PKR expression on expression of c-Fos and c-Jun protein was determined. When expression of PKR was knocked down by PKR siRNA, c-Fos and c-Jun expression were downregulated in JFH1 and H77s cells. Moreover, expression of both phosphorylated c-Fos and phosphorylated c-Jun correlated with the expression of PKR in JFH1 and H77s cells (Fig. 2E). Protein levels were quantified by normalizing to β-actin using Image J Software (Fig. S3).

Mediators upstream of c-Fos and c-Jun in the MAPK signaling pathway were assessed, and phosphorylated Erk1/2 (upstream of c-Fos) and phosphorylated JNK1 (upstream of c-Jun) were also found to positively correlate with expression of PKR (Fig. 2E), although the total expression levels of Erk1/2 and JNK1 were not changed. These results indicate that the PKR in HCC with HCV infection upregulates both the Erk1/2 to c-Fos and JNK1 to c-Jun signaling pathways. These alterations of MAPK pathways by PKR were not seen in Huh7.5.1 cells without HCV infection (Fig. 2E).

Additional confirmation that PKR positively regulates c-Fos and c-Jun signaling pathways was provided by use of the Trans AM™ Transcription Factor Assay Kit (Active Motif), which allows assessment of the DNA-binding capacity of c-Fos and c-Jun in a manner similar to the electrophoretic mobility shift assay (EMSA). Downregulation of PKR resulted in decreased DNA-binding capacity of c-Fos and c-Jun (Fig. 2F). Moreover, after the treatment by a PKR inhibitor, the expression of phosphorylated PKR was decreased, as shown by Western blotting analysis (Fig. S4). In addition, the expression of phosphorylated c-Fos and phosphorylated c-Jun was obviously decreased (Fig. S4). According to these results, we suggest that PKR would increase the phosphorylation of c-Fos and c-Jun, and then activate c-Fos and c-Jun directly.

### PKR Induces Proliferation in HCC Cells with HCV Infection that Depends on Expression of c-Fos and c-Jun

**Figure 3:**
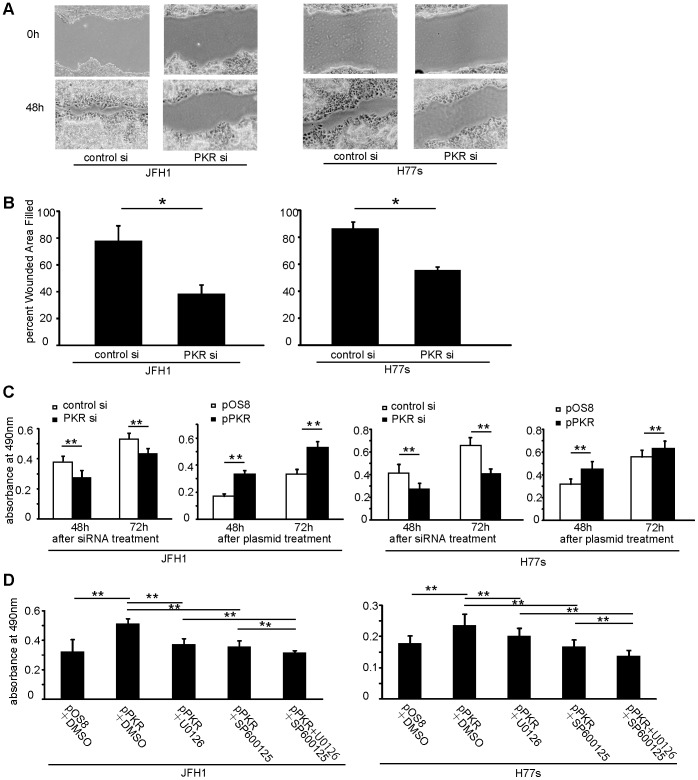
PKR expression was associated with proliferation of JFH1 and H77s cells, which was dependent on both c-Fos and c-Jun signaling pathways. Wound healing assay: Confluent monolayers of JFH1 or H77s cells transfected with PKR siRNA or control siRNA were wounded by scratching, and incubated for 48 h (A). Percent wounded area filled in with JFH1 and H77s cells (B). Mean ± SEM of six replicates. MTS assay: Proliferation was associated with PKR expression (C). Cells were transfected with pPKR, and then 20 µM c-Jun inhibitor (SP600125) and 10 µM c-Fos inhibitor (U0126) was added. MTS assay indicated that cell proliferation induced by PKR depended on both c-Jun and c-Fos pathways (D). Mean ± SEM of 10 replicates. **p<0.01, *p<0.05.

It is well known that the MAPK pathway is associated with cell proliferation [24], [25]. Therefore, assessments of proliferation were performed. Monolayer wound healing experiments revealed that JFH1 and H77s cells recovered more slowly after PKR knockdown than after transfection with control siRNA (Fig. 3A, B).

Additionally, effects on cell proliferation were evaluated by the MTS assay. Cell proliferation decreased significantly after PKR knockdown by PKR siRNA, and increased significantly when PKR was upregulated by pPKR in the JFH1 and H77s cells (p<0.01) (Fig. 3C). However these alterations of cell proliferation were not evident in Huh7.5.1 cells without HCV infection (Fig. S5).

To determine which signaling pathways are operational in cell proliferation induced by PKR, inhibition assays, using U0126 to inhibit the c-Fos pathway and SP600125 to inhibit the c-Jun pathway, were performed. We confirmed the effects of these compounds against c-Fos and c-Jun. The expression of phosphorylated c-Fos or phosphorylated c-Jun was evaluated by Western blotting after 24 h incubation with U0126 or SP600125 in both JFH1 and H77s cells (Fig. S6). The U0126 decreased phosphorylated c-Fos, but the effect on phosphorylated c-Jun was not evident in either JFH1 or H77s. SP600125 decreased phosphorylated c-Jun, but the effect on phosphorylated c-Fos was not evident. JFH1 proliferation was increased after the upregulation of PKR, and this increase was significantly diminished by either U0126 or SP600125 (p<0.05) (Fig. 3D). Use of both U0126 and SP600125 further diminished proliferation (p<0.05) to a level similar to that of JFH1 cells transfected with control plasmid (pOS8). These results indicate that both c-Fos and c-Jun signaling pathways mediate PKR-induced cell proliferation in these HCV-infected HCC cell lines.

### PKR Positively Regulates IL-8 Expression

**Figure 4:**
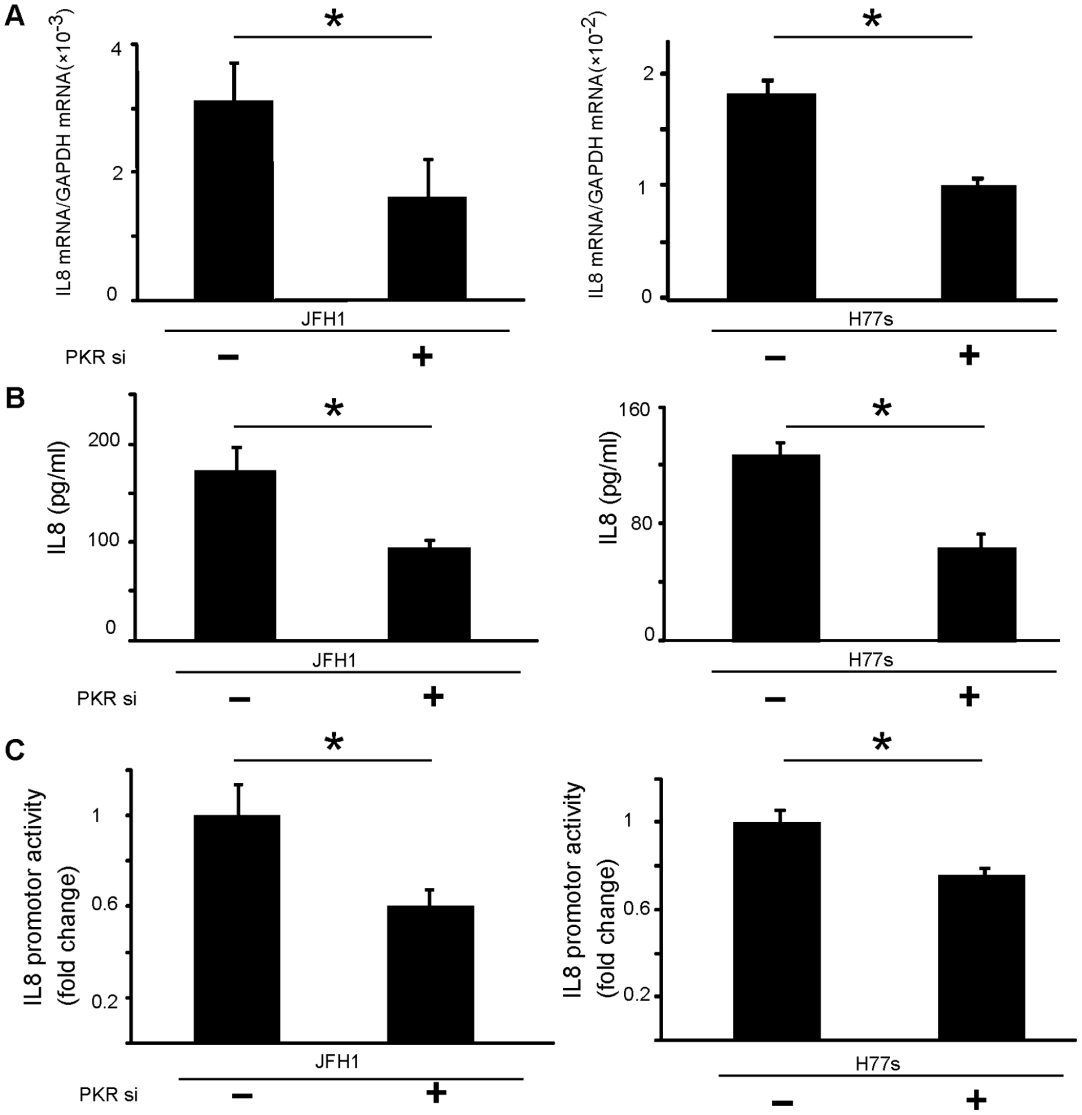
The PKR upregulates IL-8 expression and secretion. IL8 mRNA levels were downregulated by PKR knockdown (A). Supernatants of cultured cells were collected, and IL-8 levels were determined by ELISA. Secreted IL-8 was downregulated by PKR knock-down (B). Assays using the IL-8 promoter-luciferase reporter plasmid: IL-8 promoter activities were downregulated by PKR knockdown (C). Mean ± SEM of six replicates. *p<0.05.

RT-PCR revealed that IL-8 mRNA was downregulated significantly by PKR knockdown (p<0.05) (Fig. 4A), as were levels of secreted IL-8 protein in culture supernatants (Fig. 4B). IL-8 promoter activity also decreased after PKR knockdown (Fig. 4C). Thus, PKR-modulated c-Jun and c-Fos influenced production of IL-8, a molecule downstream of c-Jun and c-Fos signaling.

### c-Jun and c-Fos were Activated by PKR Expression in Human HCC Tissue with HCV Infection

The mRNA levels of c-Fos and c-Jun in 34 specimens of human HCC tissues were determined. The specimens were divided by median PKR mRNA level into two groups of higher (High PKR) and lower (Low PKR) levels. Median values for PKR mRNA in all HCC specimens, in HCC specimens with HCV infection, and in HCV-unrelated HCC specimens were 1.00×10^−3^, 1.20×10^−3^, and 8.00×10^−4^ copy ratio (PKR mRNA/GAPDH mRNA), respectively.

**Figure 5:**
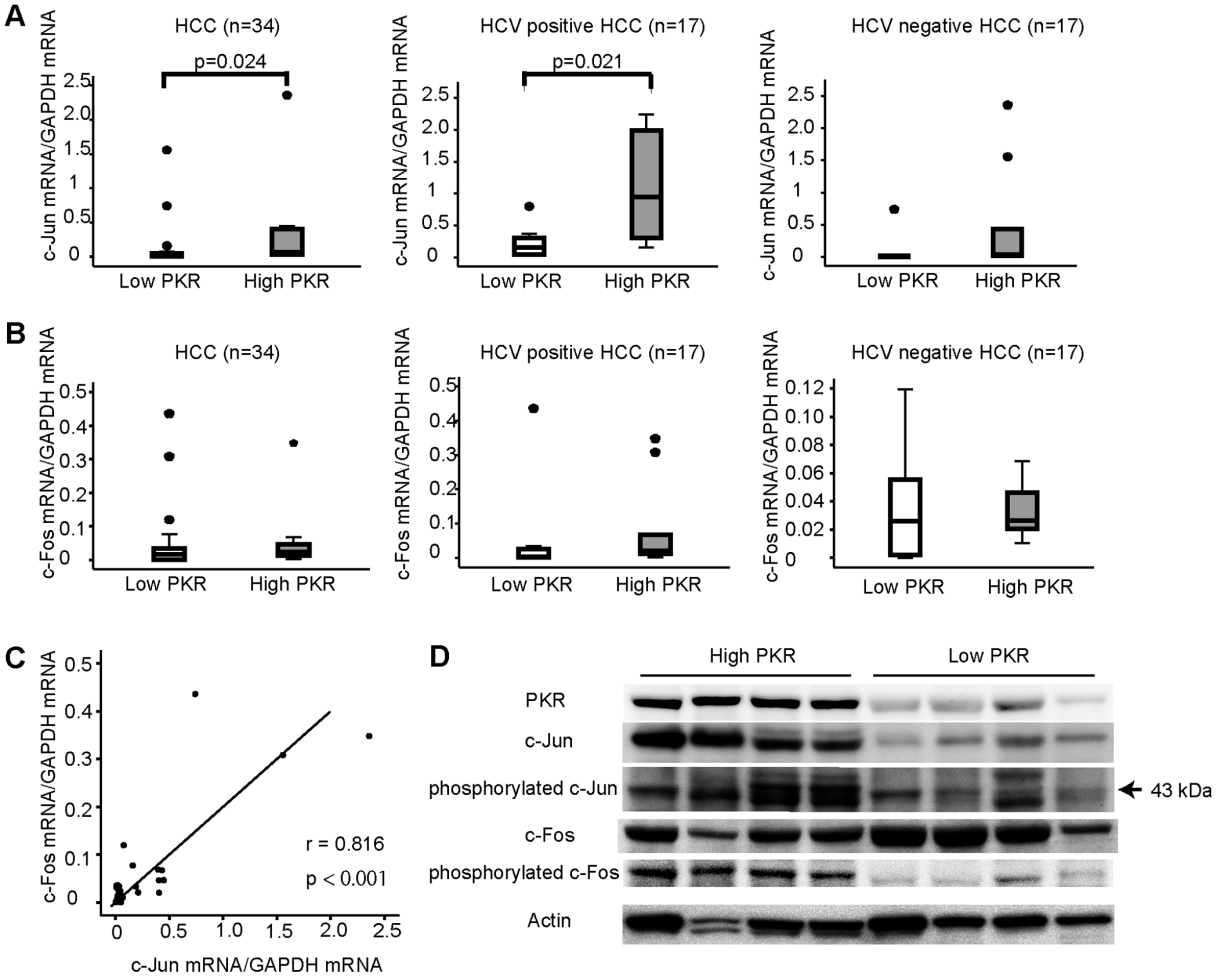
c-Jun and c-Fos were activated by the expression of PKR in human HCC tissues with HCV infection. RNA from HCC specimens of 34 patients, 17 HCC specimens with HCV infection (HCC positive HCC), and 17 HCC without HCV infection (HCC negative HCC). Each group was divided into two subsections by the median PKR mRNA values: Low PKR and High PKR. c-Jun mRNA (A) and c-Fos mRNA (B) were measured. c-Fos mRNA significantly correlated with c-Jun mRNA (r = 0.816, P<0.001) (C). The four human HCC specimens having the highest (High PKR) and the four having the lowest (Low PKR) PKR protein expression were analyzed by Western blotting. c-Jun and c-Fos in the High PKR group were activated more than in the Low PKR group (D).

When considering all 34 HCC specimens together, the High PKR Group had significantly higher levels of c-Jun mRNA than did the Low PKR group (p = 0.024). For HCC specimens with HCV infection, the c-Jun mRNA level of the High PKR group was significantly higher than that of the Low group (p = 0.021), but this difference was not evident in the HCC specimens without HCV infection (Fig. 5A). The levels of c-Fos mRNA did not differ significantly among these groups (Fig. 5B). However, a positive correlation between c-Fos and c-Jun mRNA levels was observed (r = 0.816, P<0.001) (Fig. 5C).

Additional Western blotting was performed to evaluate phosphorylated c-Jun and c-Fos levels in the human HCC specimens. The four specimens having the highest and the four having the lowest expression of PKR proteins were assayed. The specimens from the High PKR group had greater expression of phosphorylated c-Jun and phosphorylated c-Fos (Fig. 5D). These results indicate that PKR expression is related to activation of c-Jun and c-Fos in the human HCC specimens used in this study.

## Discussion

Protein kinase R (PKR) is well known as a key molecule in elimination of HCV, especially during interferon use [18]. A previously reported study demonstrated that PKR is overexpressed in HCC tissues; however, its precise role in HCC-related phenomena remains controversial. It was reported that PKR plays a tumor suppressor function through the p53 signaling pathway in human colon cancer cell lines [26]. However, other recent reports indicate that PKR can directly stimulate cell growth through the p38 MAPK and NF-κB pathways [27], [28]. The p38MAPK pathway is known to play a critical role in the pathogenesis of various malignancies [29], [30], and NF-κB is known to regulate inflammatory cytokines, growth factors, angiogenic factors and anti-apoptotic effects, each of which can contribute to the process of carcinogenesis [31]. Initially, the focus of the current study was on those molecules; however, the p53, p38MAPK and NF-κB pathways were not altered by PKR expression in the HCC cell lines with HCV infection that were used (Table S1). Moreover, results from examination of the cell cycle by FACS analysis and staining with propidium iodide revealed that the proportion of the sub-G1 population was not altered by the downregulation or upregulation of PKR (data not shown). It is possible that the role of PKR in HCC with HCV infection has a specialized function because PKR is directly induced and activated by HCV; otherwise, levels of activated PKR would be higher in this setting than in other cancers.

**Figure 6:**
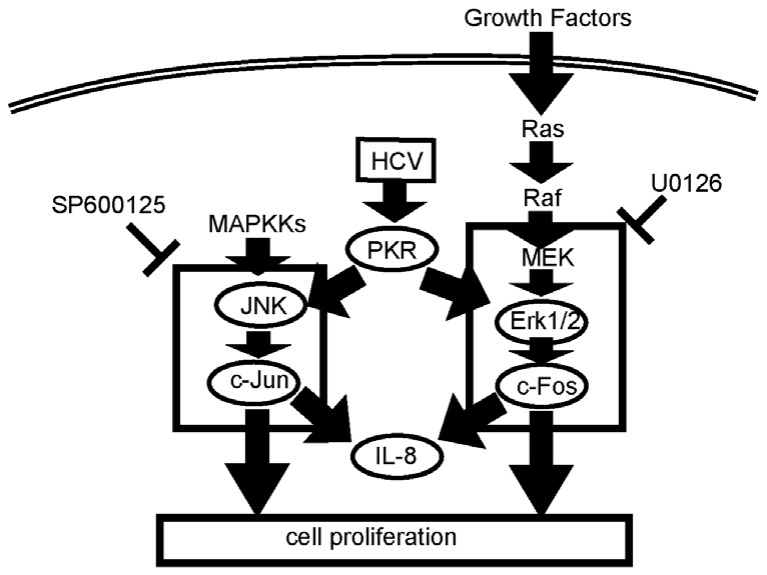
Model of the role of PKR in cell proliferation and relationships between PKR and other molecules associated with the c-Jun and c-Fos pathways. SP600125: inhibitor of c-Jun pathway. U0126: inhibitor of c-Fos pathway.

This study demonstrated that PKR is more highly activated in HCC cells with HCV infection, and that under those conditions, the over-activated PKR upregulates c-Jun and c-Fos through activation of JNK1 and p44/42 MAPK (Erk1/2), respectively (Fig. 6). Upregulation of c-Jun and c-Fos was dependent on tumor cell proliferation, and in turn induced IL-8. Activation of c-Jun and c-Fos was also observed in the human HCC specimens, in which PKR was overexpressed. The functions of over-activated PKR would be expected to have negative effects on prognosis for HCC patients with HCV infection, and could be related to the worse outcomes observed in this setting.

c-Jun and c-Fos are members of the activating protein 1 (AP-1) family. These proteins are diametric transcription factors [32] that bind to DNA [33]. The AP-1 family is implicated in carcinogenesis, and it regulates genes that include important regulators of invasion and metastasis, proliferation, differentiation, and survival [34]. Among the AP-1 proteins, c-Jun is able to both homo- and heterodimerize, whereas c-Fos is unable to homodimerize. In vitro studies have shown that Jun-Fos heterodimers are more stable and have stronger DNA-binding activity than Jun-Jun homodimers [35]. The current results in human HCC tissues indicated a positive correlation between levels of c-Fos and c-Jun (Figure 6C), suggesting that c-Jun and c-Fos hetero-dimers could be the main moieties binding to DNA in HCC.

Previous reports indicated that c-Jun is important for hepatogenesis [36], [37]. In mice with inactivated c-Jun, the number and size of hepatic tumors caused by diethylnitrosamine (DEN) treatment were reduced [38], suggesting that c-Jun may have an important role in tumor progression. On the other hand, persistent expression of c-Fos would also play an important role in tumor progression. c-Fos is known to induce depolarization and acquisition of an invasive phenotype, a process generally referred to as “epithelial to mesenchymal transition” (EMT) [39], resulting in tumor growth and metastasis. In a previous study of 150 human HCC samples, c-Fos expression was significantly higher in tumors than in non-tumor tissue (p<0.0001) [40], and elevated JNK1 (the c-Jun upstream activator) activity was identified in more than 50% of HCC samples relative to non-cancerous liver tissue [41]. In the current study, upregulation of c-Jun was identified in HCC tissues with higher expression of PKR. These findings suggest that alterations of c-Jun and c-Fos pathways in HCC with HCV infection may have important roles in tumor progression.

The direct effects of HCV on the c-Jun and c-Fos pathways must be considered. A previous report suggests that HCV proteins can modulate MAPK (Erk) signaling by targeting multiple steps along the signaling pathways [42]. Another report indicated that HCV E2 protein activates the MAPK (Erk) pathway and promotes cell proliferation in human hepatoma Huh-7 cells [42]. The current results suggest that PKR may activate Erk1/2 directly through phosphorylation. The previous reports did not describe PKR expression, and it is possible HCV proteins modulate the MAPK (Erk) pathway through the activation of PKR. On the other hand, one previous report described negative regulation of Erk by PKR, but positive regulation of JNK by PKR in mouse embryonic stem cells [43]. HCV infection may alter the role of PKR with respect to the c-Jun and c-Fos pathways: differences in this role in HCC as compared to other cancer types need to be determined in future studies. Recently, the multikinase inhibitor, sorafenib, was reported to be effective for treatment of HCC [44]. Sorafenib was shown to inhibit c-Raf, Erk and MAPK/Erk kinase (MEK) [44], and therefore would have a greater effect in HCC patients with HCV. The role of PKR in treatment with sorafenib should be addressed in future analyses.

Over-activated PKR in HCC with HCV infection upregulated production of IL-8 in this study. IL-8 occurs downstream to AP-1 [45], and is known to contribute to angiogenesis and proliferation [46] in various cancers. It is known that HCV NS5A can induce IL-8 mRNA and protein [47], and in a previous report, serum levels of IL-8 were increased significantly in patients with chronic hepatitis C compared with normal controls [48]. Activated PKR could contribute to the upregulation of IL-8 in patients with HCV, which could in turn contribute to tumor progression. The precise role of upregulated IL-8 in HCC with HCV infection also needs to be ascertained in detail in future studies.

In conclusion, the current results demonstrate that over-activated PKR in HCC with HCV infection contributes to proliferation due to the upregulation of c-Jun and c-Fos by activation of JNK and Erk1/2. Such effects of over-activated PKR would be expected to have negative effects on treatment of HCC patients with HCV infection. The role of PKR and its importance for elimination of HCV in patients with HCC needs to be reconsidered. Over-activated PKR potentially could serve as a therapeutic target in HCC patients with HCV infection; further studies are warranted to investigate this possibility.

## Supporting Information

Figure S1 **HCV infection upregulates the expression of phosphorylated PKR protein.** 24 h after transfection of Huh 7.5.1cells with pJFH1-full or pH77s-full, protein expression of PKR and phosphorylated PKR were determined by Western blotting. In the HCV-infected HCC cells (JFH1 and H77s), expression of phosphorylated PKR was increased significantly compared with that in Huh7.5.1 cells (HCV-uninfected HCC cells).
(TIF)

Figure S2 **Quantification of** **Figure 1E** **Western blot bands.** Bands in Figure 1E indicating PKR, phosphorylated PKR and phosphorylated eIF2α were quantified and normalized using the quantified corresponding beta-actin band.
(TIF)

Figure S3 **Quantification of** **Figure 2E** **Western blot bands.** Bands in Figure 2E indicating c-Fos, c-Jun, phosphorylated c-Fos and phosphorylated c-Jun were quantified and normalized using the quantified corresponding beta-actin band.
(TIF)

Figure S4 **PKR inhibitor decreased phosphorylation of c-Fos and c-Jun.** PKR inhibitor (300 nM) was added to JFH1 and H77s. After a 24 h treatment, the expression of PKR, c-Fos, c-Jun, phosphorylated PKR, phosphorylated c-Fos, and phosphorylated c-Jun were evaluated by Western blotting. PKR inhibitor decreased phosphorylated PKR, and phosphorylated c-Jun and phosphorylated c-Fos proteins.
(TIF)

Figure S5 **Enhancement of cell proliferation was not evident in Huh7.5.1 cells without HCV infection.** Wound healing assay: Confluent monolayers of Huh7.5.1 cells transfected with PKR siRNA or control siRNA were wounded by scratching and then incubated for 48 h (A). Percent wounded area filled in with Huh7.5.1 cells. Mean ± SEM of six replicates (B). MTS assay: Proliferation was not associated with PKR expression in Huh7.5.1 (C). Mean ± SEM of 10 replicates.
(TIF)

Figure S6 **U0126 inhibits c-Fos signaling pathway, and SP600125 inhibits c-Jun signaling pathway, in JFH1 and in H77s cells.** 10 µM c-Fos inhibitor (U0126) or 20 µM c-Jun inhibitor (SP600125) was added to JFH1 and H77s cell cultures. After a 24 h treatment, levels of phosphorylated c-Fos and phosphorylated c-Jun were evaluated by Western blotting. U0126 decreased phosphorylated c-Fos, and SP600125 decreased phosphorylated c-Jun.
(TIF)

Table S1 PCR array analysis of the effect of PKR up- and down-regulation on cancer-related genes.
(DOC)
