# Supplementary figures and images for: Protein Kinase R Modulates c-Fos and c-Jun Signaling to Promote Proliferation of Hepatocellular Carcinoma with Hepatitis C Virus Infection
Source: PLoS One. 2013 Jul 2;8(7):e67750. doi: 10.1371/journal.pone.0067750 (PMC3699507; doi:10.1371/journal.pone.0067750)

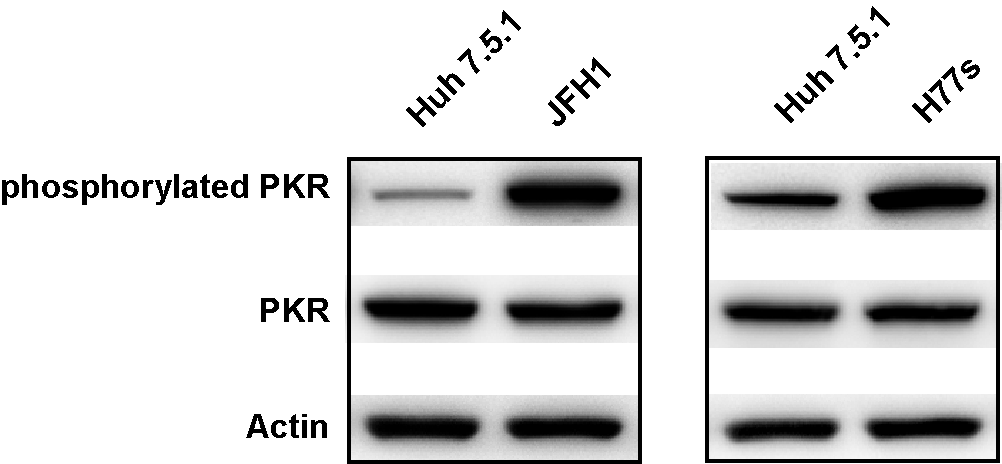

Supplement: Figure S1 — HCV infection upregulates the expression of phosphorylated PKR protein. 24 h after transfection of Huh 7.5.1cells with pJFH1-full or pH77s-full, protein expression of PKR and phosphorylated PKR were determined by Western blotting. In the HCV-infected HCC cells (JFH1 and H77s), expression of phosphorylated PKR was increased significantly compared with that in Huh7.5.1 cells (HCV-uninfected HCC cells). (TIF) [file pone.0067750.s001.tif]

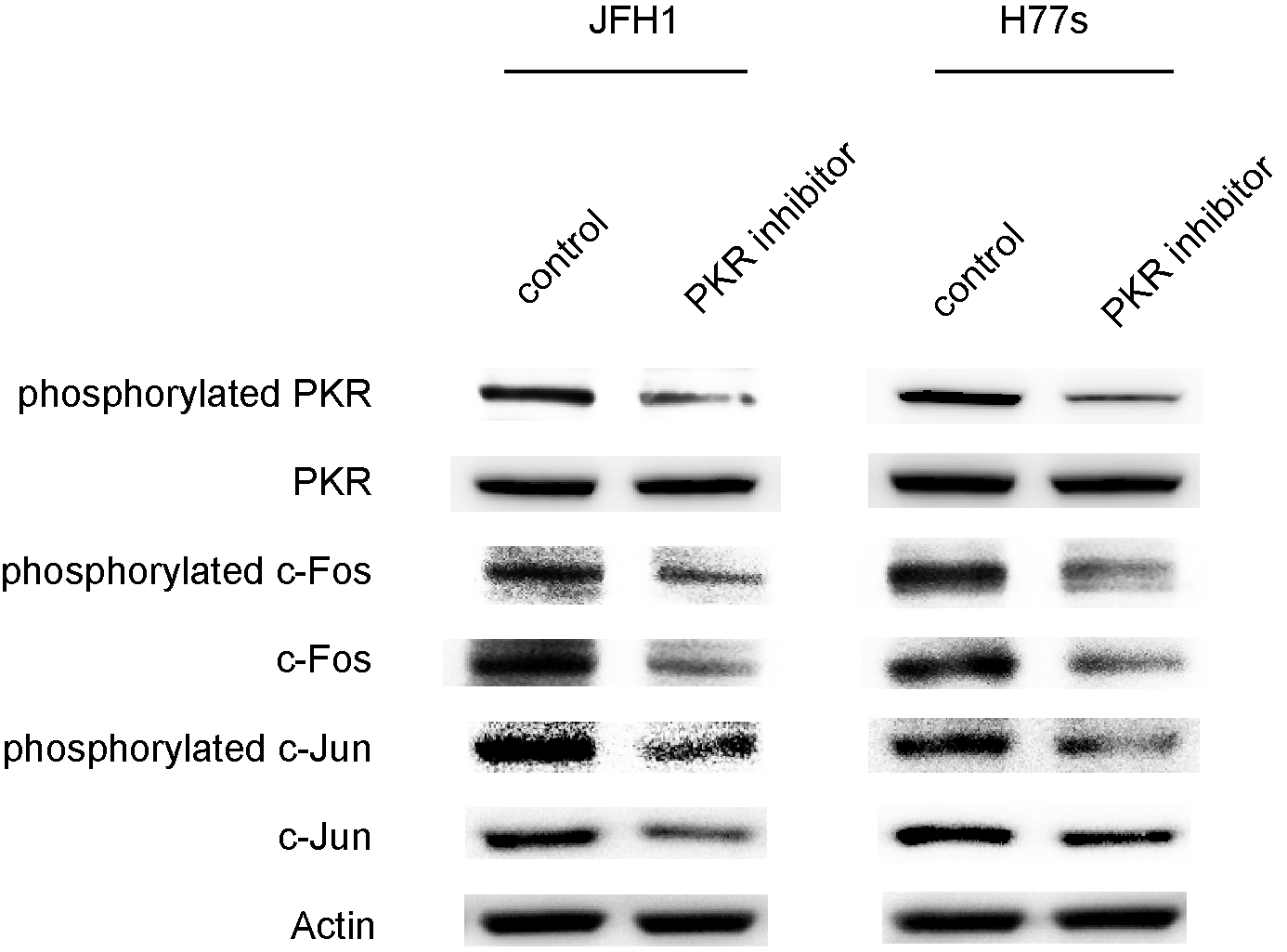

Supplement: Figure S4 — PKR inhibitor decreased phosphorylation of c-Fos and c-Jun. PKR inhibitor (300 nM) was added to JFH1 and H77s. After a 24 h treatment, the expression of PKR, c-Fos, c-Jun, phosphorylated PKR, phosphorylated c-Fos, and phosphorylated c-Jun were evaluated by Western blotting. PKR inhibitor decreased phosphorylated PKR, and phosphorylated c-Jun and phosphorylated c-Fos proteins. (TIF) [file pone.0067750.s004.tif]

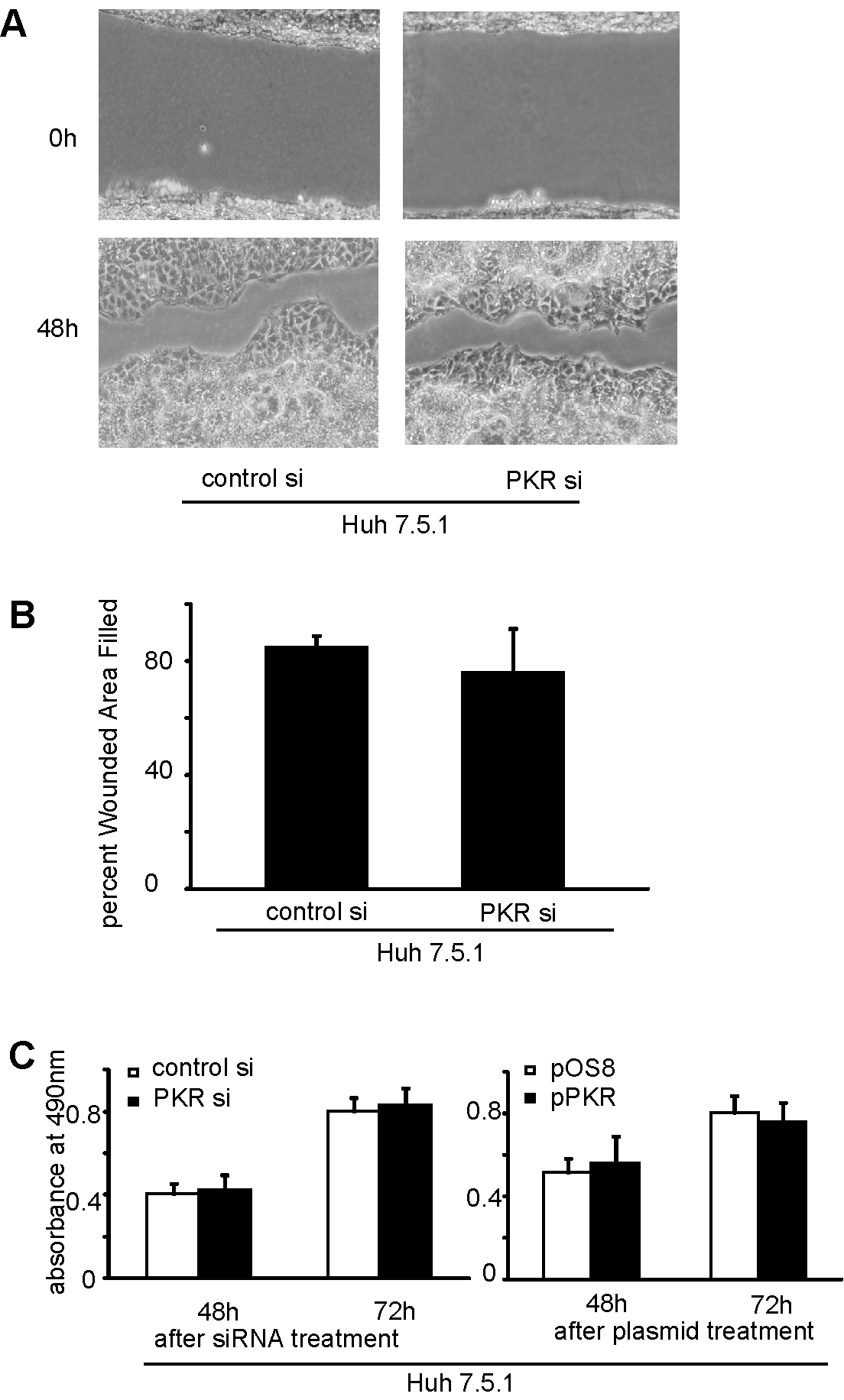

Supplement: Figure S5 — Enhancement of cell proliferation was not evident in Huh7.5.1 cells without HCV infection. Wound healing assay: Confluent monolayers of Huh7.5.1 cells transfected with PKR siRNA or control siRNA were wounded by scratching and then incubated for 48 h (A). Percent wounded area filled in with Huh7.5.1 cells. Mean ± SEM of six replicates (B). MTS assay: Proliferation was not associated with PKR expression in Huh7.5.1 (C). Mean ± SEM of 10 replicates. (TIF) [file pone.0067750.s005.tif]

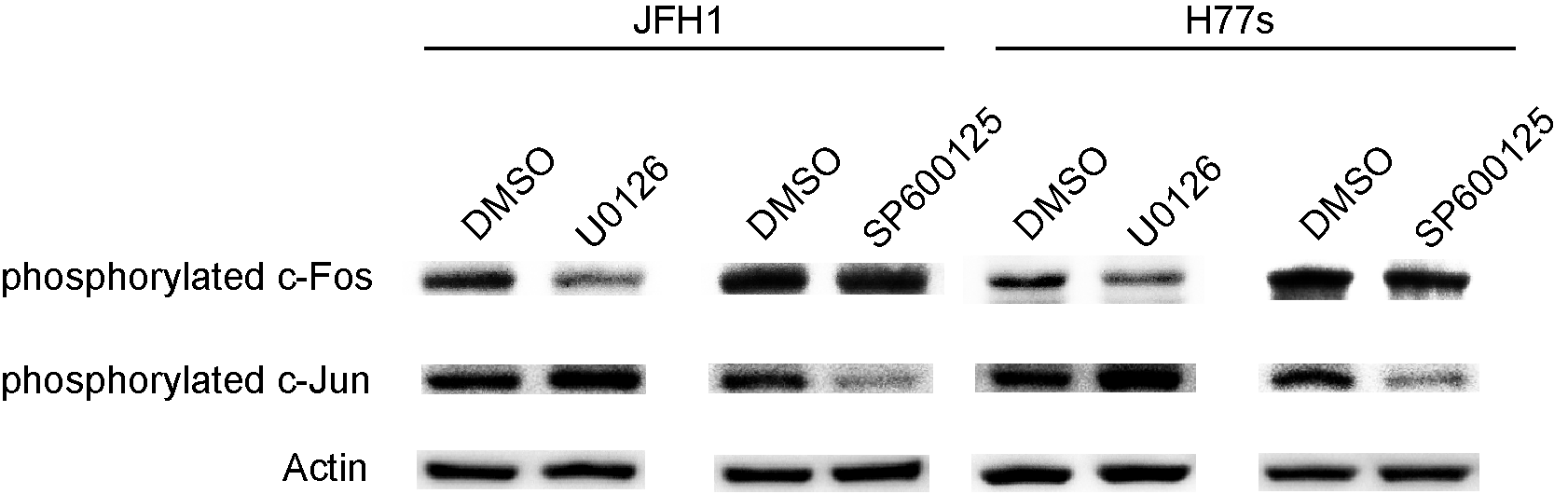

Supplement: Figure S6 — U0126 inhibits c-Fos signaling pathway, and SP600125 inhibits c-Jun signaling pathway, in JFH1 and in H77s cells. 10 µM c-Fos inhibitor (U0126) or 20 µM c-Jun inhibitor (SP600125) was added to JFH1 and H77s cell cultures. After a 24 h treatment, levels of phosphorylated c-Fos and phosphorylated c-Jun were evaluated by Western blotting. U0126 decreased phosphorylated c-Fos, and SP600125 decreased phosphorylated c-Jun. (TIF) [file pone.0067750.s006.tif]
