# Supplementary material for: Protein Kinase R Modulates c-Fos and c-Jun Signaling to Promote Proliferation of Hepatocellular Carcinoma with Hepatitis C Virus Infection
Source: PLoS One. 2013 Jul 2;8(7):e67750. doi: 10.1371/journal.pone.0067750 (PMC3699507; doi:10.1371/journal.pone.0067750)
Supplement: Table S1 — PCR array analysis of the effect of PKR up- and down-regulation on cancer-related genes. (DOC) [file pone.0067750.s007.doc]

Supporting Table 1

PCR array analysis of the effect of PKR up- and down-regulation on cancer-related genes

| Gene | Huh 7.5.1 | | JFH1 | |  | Gene | Huh 7.5.1 | | JFH1 | |
| --- | --- | --- | --- | --- | --- | --- | --- | --- | --- | --- |
|  | PKR siRNA  /control siRNA | pOS8/  pPKR | PKRsiRNA  /control siRNA | pOS8/  pPKR |  |  | PKR siRNA  /control siRNA | pOS8/  pPKR | PKR siRNA  /control siRNA | pOS8/  pPKR |
| AKT1 | -1.06 | 1.02 | 1.91 | -1.03 |  | MET | -1.46 | -1.27 | -1.80 | 1.33 |
| ANGPT1 | -1.03 | N/A | -1.19 | 1.15 |  | MMP1 | -1.03 | -1.00 | -1.19 | 1.15 |
| ANGPT2 | -2.69 | -1.08 | -1.53 | 1.06 |  | MMP2 | -1.03 | 1.64 | -1.19 | 1.15 |
| APAF1 | 1.15 | -1.97 | -1.03 | 1.25 |  | MMP9 | 3.14 | 1.65 | 1.70 | -1.25 |
| ATM | -1.03 | -1.41 | -1.32 | 1.44 |  | MTA1 | -1.23 | 1.08 | 2.79 | -1.75 |
| BAD | 1.02 | -1.46 | -1.21 | -1.47 |  | MTA2 | 1.04 | -1.03 | 1.19 | 1.29 |
| BAX | -1.42 | 1.12 | -1.20 | 1.08 |  | MTSS1 | -2.53 | 1.10 | -1.15 | 1.16 |
| BCL2 | 1.22 | 3.96 | 1.92 | 1.29 |  | MYC | 1.20 | -4.78 | -1.44 | -2.43 |
| BCL2L1 | -1.42 | -1.61 | -1.97 | 1.78 |  | NFKB1 | 1.27 | 1.05 | -1.28 | -1.38 |
| BRCA1 | 1.17 | -1.75 | 1.06 | 1.17 |  | NFKBIA | 1.13 | -1.97 | -1.19 | -3.11 |
| CASP8 | -1.35 | -1.08 | -1.14 | -1.26 |  | NME1 | -2.20 | -1.03 | 1.02 | -1.03 |
| CCNE1 | 1.02 | 1.07 | 1.05 | 1.13 |  | NME4 | 1.26 | -1.95 | -1.01 | -1.06 |
| CDC25A | -1.24 | 1.03 | 1.92 | -1.66 |  | PDGFA | -1.31 | -1.68 | -1.64 | -1.12 |
| CDK2 | 1.04 | -1.13 | 1.15 | -1.20 |  | PDGFB | -1.27 | 1.01 | -1.33 | 1.70 |
| CDK4 | -2.48 | 1.04 | -1.94 | 1.15 |  | PIK3R1 | 1.06 | 1.17 | -1.03 | 1.64 |
| CDKN1A | -1.04 | -2.67 | -1.52 | 1.03 |  | PLAU | 1.19 | 1.02 | 1.63 | 1.93 |
| CDKN2A | 1.22 | -1.10 | -1.15 | 1.23 |  | PLAUR | -1.21 | 1.49 | 1.77 | -1.64 |
| CFLAR | 1.16 | -2.40 | -1.26 | -2.86 |  | PNN | -1.06 | -1.08 | 1.44 | -1.18 |
| CHEK2 | -1.56 | -1.07 | 1.05 | -1.32 |  | RAF1 | -1.88 | -1.04 | -1.58 | 1.03 |
| COL18A1 | 1.03 | -2.74 | -1.51 | -1.50 |  | RB1 | -1.05 | -3.11 | -1.17 | -1.71 |
| E2F1 | -1.43 | -1.85 | -2.20 | 1.19 |  | S100A4 | 1.40 | 1.02 | 1.17 | -1.34 |
| ERBB2 | 1.07 | -1.52 | -1.10 | 1.01 |  | SERPINB5 | 1.17 | 9.81 | 2.50 | 3.46 |
| ETS2 | -1.03 | -1.16 | -1.26 | 1.17 |  | SERPINE1 | -2.14 | -1.05 | -1.59 | 1.06 |
| FAS | 1.13 | -1.42 | 1.86 | -2.82 |  | SNCG | 1.50 | 5.71 | 1.37 | 2.24 |
| FGFR2 | -1.03 | -1.00 | N/A | 1.15 |  | SYK | 2.59 | 4.33 | 2.77 | 2.48 |
| FOS | -1.05 | 1.28 | -1.46 | 1.47 |  | TEK | -1.03 | -1.00 | -1.19 | 1.15 |
| GZMA | -1.03 | 1.45 | -1.19 | -1.10 |  | TERT | -1.37 | -1.11 | -1.32 | 2.13 |
| HTATIP2 | 1.17 | -3.13 | -1.16 | -1.18 |  | TGFB1 | 1.31 | 1.33 | 1.00 | 2.09 |
| IFNA1 | -1.03 | -1.00 | -1.19 | 1.15 |  | TGFBR1 | -1.20 | 1.35 | 1.36 | -1.49 |
| IFNB1 | -1.03 | 4.15 | -1.52 | 1.15 |  | THBS1 | -1.19 | -1.05 | -1.32 | 1.09 |
| IGF1 | -2.38 | -1.01 | -1.27 | -1.06 |  | TIMP1 | -1.46 | 3.28 | 1.12 | 1.11 |
| IL8 | -1.03 | -1.97 | -2.11 | -1.26 |  | TIMP3 | 1.02 | -2.60 | -1.18 | -1.55 |
| ITGA1 | -1.20 | -1.46 | -1.39 | 1.36 |  | TNF | -1.03 | -1.00 | -1.19 | 1.15 |
| ITGA2 | -1.08 | -1.43 | -1.55 | 1.11 |  | TNFRSF10B | 1.10 | -1.34 | 1.05 | 1.07 |
| ITGA3 | 1.29 | 3.72 | 2.27 | 2.16 |  | TNFRSF1A | -1.43 | -1.21 | -1.20 | -1.01 |
| ITGA4 | -1.12 | 1.65 | -1.14 | -1.81 |  | TNFRSF25 | 1.20 | 2.23 | 1.55 | 3.82 |
| ITGAV | -1.36 | 1.29 | 1.45 | -1.89 |  | TP53 | -1.41 | -1.15 | -1.20 | 1.31 |
| ITGB1 | -1.09 | -1.01 | -1.01 | -1.23 |  | TWIST1 | -1.03 | -1.00 | -1.19 | 1.15 |
| ITGB3 | -1.37 | 2.49 | -1.35 | 1.55 |  | EPDR1 | 1.05 | -1.04 | -1.66 | 1.53 |
| ITGB5 | 1.10 | -4.37 | -1.28 | -1.07 |  | VEGFA | 1.17 | 1.26 | 1.04 | 2.15 |
| JUN | 1.02 | 1.01 | -1.36 | 1.55 |  | B2M | -1.05 | 1.47 | -1.04 | -1.16 |
| MAP2K1 | 1.00 | -2.24 | -1.19 | -2.67 |  | HPRT1 | -1.05 | -1.08 | 1.29 | -1.01 |
| MCAM | -1.97 | -1.11 | 1.06 | -1.02 |  | RPL13A | 1.07 | 1.11 | 1.01 | 1.04 |
| MDM2 | -1.20 | -3.47 | -1.21 | -1.29 |  |  |  |  |  |  |

Samples for PCR array analysis were generated by mixing 4 RNA samples.
